# Target specific inhibition of West Nile virus envelope glycoprotein and methyltransferase using phytocompounds: an *in silico* strategy leveraging molecular docking and dynamics simulation

**DOI:** 10.3389/fmicb.2023.1189786

**Published:** 2023-06-28

**Authors:** Shopnil Akash, Imren Bayıl, Md. Anisur Rahman, Nobendu Mukerjee, Swastika Maitra, Md. Rezaul Islam, Sanchaita Rajkhowa, Arabinda Ghosh, Sami A. Al-Hussain, Magdi E. A. Zaki, Vikash Jaiswal, Sanjit Sah, Joshuan J. Barboza, Ranjit Sah

**Affiliations:** ^1^Department of Pharmacy, Faculty of Allied Health Sciences, Daffodil International University, Dhaka, Bangladesh; ^2^Department of Bioinformatics and Computational Biology, Gaziantep University, Gaziantep, Türkiye; ^3^Department of Pharmacy, Islamic University, Kushtia, Bangladesh; ^4^Department of Microbiology, West Bengal State University, Kolkata, West Bengal, India; ^5^Department of Health Sciences, Novel Global Community Educational Foundation, Hebersham, NSW, Australia; ^6^Department of Microbiology, Adamas University, Kolkata, West Bengal, India; ^7^Centre for Biotechnology and Bioinformatics, Dibrugarh University, Dibrugarh, Assam, India; ^8^Microbiology Division, Department of Botany, Gauhati University, Gwahati, Assam, India; ^9^Department of Chemistry, Imam Mohammad Ibn Saud Islamic University (IMSIU), Riyadh, Saudi Arabia; ^10^Department of Cardiovascular Research, Larkin Community Hospital, South Miami, FL, United States; ^11^Global Consortium for Public Health and Research, Datta Meghe Institute of Higher Education and Research, Jawaharlal Nehru Medical College, Wardha, India; ^12^SR Sanjeevani Hospital, Kayanpur, Siraha, Nepal; ^13^Escuela de Medicina, Universidad Cesar Vallejo, Trujillo, Peru; ^14^Tribhuvan University Teaching Hospital, Institute of Medicine, Kathmandu, Nepal; ^15^Dr. D. Y. Patil Medical College, Hospital and Research Centre, Dr. D. Y. Patil Vidyapeeth, Pune, Maharashtra, India; ^16^Department of Public Health Dentistry, Dr. D.Y. Patil Dental College and Hospital, Dr. D.Y. Patil Vidyapeeth, Pune, Maharashtra, India

**Keywords:** West Nile virus, molecular docking, molecular dynamic simulation, PCA, drug-likeness, ADMET

## Abstract

Mosquitoes are the primary vector for West Nile virus, a flavivirus. The virus’s ability to infiltrate and establish itself in increasing numbers of nations has made it a persistent threat to public health worldwide. Despite the widespread occurrence of this potentially fatal disease, no effective treatment options are currently on the market. As a result, there is an immediate need for the research and development of novel pharmaceuticals. To begin, molecular docking was performed on two possible West Nile virus target proteins using a panel of twelve natural chemicals, including Apigenin, Resveratrol, Hesperetin, Fungisterol, Lucidone, Ganoderic acid, Curcumin, Kaempferol, Cholic acid, Chlorogenic acid, Pinocembrin, and Sanguinarine. West Nile virus methyltransferase (PDB ID: 2OY0) binding affinities varied from −7.4 to −8.3 kcal/mol, whereas West Nile virus envelope glycoprotein affinities ranged from −6.2 to −8.1 kcal/mol (PDB ID: 2I69). Second, substances with larger molecular weights are less likely to be unhappy with the Lipinski rule. Hence, additional research was carried out without regard to molecular weight. In addition, compounds 01, 02, 03, 05, 06, 07, 08, 09, 10 and 11 are more soluble in water than compound 04 is. Besides, based on maximum binding affinity, best three compounds (Apigenin, Curcumin, and Ganoderic Acid) has been carried out molecular dynamic simulation (MDs) at 100 ns to determine their stability. The MDs data is also reported that these mentioned molecules are highly stable. Finally, advanced principal component analysis (PCA), dynamics cross-correlation matrices (DCCM) analysis, binding free energy and dynamic cross correlation matrix (DCCM) theoretical study is also included to established mentioned phytochemical as a potential drug candidate. Research has indicated that the aforementioned natural substances may be an effective tool in the battle against the dangerous West Nile virus. This study aims to locate a bioactive natural component that might be used as a pharmaceutical.

## 1. Introduction

The infectious disease has the potential to cause a global pandemic and has been transmitted regularly throughout human history, such as plague, cholera, the flu, SARS-CoV, and MERS-CoV (Fahmi et al., 2021; Piret and Boivin, 2021). Besides, many infectious diseases may lead to pandemics, including Monkeypox, and Marburg virus (Farahat and Memish, 2022). These infections were introduced to humans due to increased interaction with animals due to activities such as breeding, hunting, and global commerce. Recently, another new viral disease has been affecting people all over the globe, whose name is the West Nile virus. In 1937, researchers discovered the West Nile virus for the first time on the African continent, specifically in the West Nile area of Uganda (Sule et al., 2018). Before 1997, the West Nile virus was not thought to be harmful to birds. However, in Israel around that time, a more dangerous strain of the virus was responsible for the deaths of many bird species with symptoms of encephalitis and paralysis. Since the beginning of the 1950s, several nations worldwide have reported cases of human infections caused by the West Nile virus (Hayes, 2001). Today, the West Nile virus is the most common infectious disease spread by mosquitoes on the American continent. It is transmitted from person to person, most often by the bite of an infected mosquito. The West Nile virus infection was reported in two persons in New York City on August 16, 2022, one in Brooklyn and another in Queens. According to an announcement made by the New York City Health Department, the virus was also identified in a “record number” of infected mosquitoes around the city. According to the declaration made by the health authority, there have been a minimum of 54 instances detected throughout the continent this year, along with four fatalities. The risk of contracting the West Nile virus is highest during the mosquito season, which begins in the summer and lasts into the autumn (New York Times, 2022).

The majority of people who have been diagnosed with the West Nile virus do not experience any symptoms of illness. Fever and other symptoms like headaches, body aches, joint pains, diarrhea, vomiting, or a rash arise in around one of every five infected patients. About one in every 150 infected persons may get a severe disease, which can often be deadly. Over the age of 60, people have the greatest chance of developing this life-threatening disease. Positive cases in New York City had a median age of 62. Those who already have preexisting health problems, such as cancer, diabetes, or hypertension, may be at a greater risk of developing severe further health issues. In extreme cases of West Nile virus, recovering might require several weeks or months, but impairment to the central nervous system can be incurable (New York Times, 2022).

The West Nile virus infection should be included in the differential diagnosis, and particular laboratory test findings should raise a high index of clinical suspicion. The IgM-capture enzyme-linked immunosorbent test is the most often applied diagnostic technique. This test has a sensitivity range of 95 to 100% in serum and spinal fluid. The IgM is usually detected in the serum and cerebrospinal fluid by the time the sickness manifests itself. When a person has encephalitis or meningitis, a high IgM of West Nile virus antibodies is undoubtedly indicative of infection; however, the IgM may last for many months to more than a year because humoral IgM antibodies do not penetrate the blood–brain barrier, an intrathecal West Nile virus-specific IgM strongly supports a central nervous system infection (Kemmerly, 2003).

The clinical care of West Nile virus diseases is considered supportive since neither vaccination nor any drug can be used to treat or prevent West Nile virus in humans. Patients experiencing severe meningeal symptoms typically require pain relief for their headaches, antiemetic treatment, and rehydration for the nausea and vomiting associated with their condition (CDC, n.d.).

Although, a huge number of people are affecting in recent time, but no authorized medication or potential vaccine is available still to fight the West Nile virus (Bergmann et al., 2022). Therefore, it is of the utmost importance issues to rapidly advance the research and development of potent anti-viral medications to combat this lethal pathogenic viral infection.

To development of a novel drug candidate, required more than 10 years of times, huge amount of money and resources (Rahman et al., 2012). So, the primary goal of *in silico* research is to develop a drug design strategy to predict the inhibitory activity of a series of natural compounds against West Nile virus with the help of advanced *in silico* method such as molecular docking, molecular dynamics simulations, ADMET, principal component analysis (PCA), dynamics cross-correlation matrices (DCCM) analysis, binding free energy and dynamic cross correlation matrix (DCCM). The molecular docking studies was conducted to determine the binding affinity of the selected phytocompounds with the target proteins of the West Nile virus and then based on maximum affinity, three most potential compounds were carried out to the molecular dynamics simulations and determine the stability and binding energies of the protein-ligand complexes. Ultimately the results of this investigation have the potential to contribute to the development of novel drugs for the treatment of West Nile virus infection.

## 2. Literature based evidence

### 2.1. Pharmacological activity of reported compounds in earlier studies and based on which compound are chosen

#### 2.1.1. Apigenin pharmacological activity

Apigenin (APG) is a flavonoid found in high concentrations in many fruits, vegetables, and Chinese medicinal herbs. It has a wide range of physiological effects, including anti-inflammatory, antioxidant, antibacterial, anti-viral, and blood pressure-lowering properties (Yan et al., 2017). Apigenin has been shown to have anti-cancer, anti-diabetic, and antioxidant effects. APG inhibits tumor cell growth and survival by triggering apoptosis, cell cycle arrest, and the creation of ROS. It also prevents metastasis and angiogenesis by changing various cellular signaling pathways (Zhou et al., 2022). APG suppresses Enterovirus-71 infection by interfering with RNA-transacting factor interactions (Zhang et al., 2014).

#### 2.1.2. Resveratrol antiviral activity

Resveratrol is a natural chemical produced by certain plants (Campagna and Rivas, 2010). Resveratrol has anti-viral activity against human and animal viruses (Abba et al., 2015). Resveratrol has also inhibitory effects on various viruses (Zhao et al., 2017).

#### 2.1.3. Hesperetin pharmacological activity

Hesperetin is a flavanone, and in its water-soluble glycoside form of hesperidin, it can be found in various citrus fruits. Hesperetin has shown anti-viral properties *in vitro* against a few RNA viruses (Zandi et al., 2011). Hesperidin also demonstrated anti-viral activity against the growth of the 17D strain of the yellow fever virus (Agrawal et al., 2021). The bioflavonoid Hesperetin inhibits the intracellular reproduction of viruses (Oo et al., 2016).

#### 2.1.4. Fungisterol anti-viral activity

Fungisterol, a compound found in mushrooms, has anti-viral properties (Zhu et al., 2021). Potential anti-viral effects of Fungisterol have been demonstrated against various pathogens, including the human immunodeficiency virus (HIV), influenza, herpes simplex virus (HSV), hepatitis B and C viruses, and others (Raut and Adhikari, 2021).

#### 2.1.5. Lucidone pharmacological activity

Lucidone is a natural compound found in the fruiting body of the edible mushroom Ganoderma lucidum. It belongs to the class of organic compounds known as coumarins, which are fragrant and sweet-smelling organic compounds that are commonly found in plants (Boh et al., 2007). Recent research has been reported that the Lucidone might be composed to have various pharmacological properties, including antioxidant, anti-inflammatory, antitumor, antiviral and neuroprotective activities. It also reported that the Lucidone suppresses dengue viral replication through the induction of heme oxygenase-1 and suppresses Hepatitis C Virus (Chen et al., 2013, 2018).

#### 2.1.6. Ganoderic acid anti-viral activity

Ganoderic acid (GA) has been shown to have anti-viral, antihypertensive, anti-cancer, and immune-modulatory effects (Zhu et al., 2021). Anti-viral effects of Ganoderic acid against Enterovirus 71 (EV71) infection are observed without cytotoxicity (Zhang et al., 2014). There is evidence that Ganoderic acid can inhibit viral replication and reduce the extent of liver damage (Li and Wang, 2006).

#### 2.1.7. Curcumin pharmacological activity

It has also been demonstrated that Curcumin prevents the development of several viruses and cancerous cells. Curcumin can block the production of HSV-1’s immediate early genes and the action of the HIV-1 integrase, both of which are required for the virus’s replication (Zandi et al., 2010). Through mechanisms present in the infected cells, Curcumin can stop the production of viral genes and, consequently, viral replication (Kutluay et al., 2008).

#### 2.1.8. Kaempferol pharmacological activity

Previous reports indicate flavanol Kaempferol and its glycosides have strong anti-viral properties (Khazdair et al., 2021). Kaempferol could prevent the spread of viruses in the brain, lungs, kidneys, heart, and spleen. According to research, the hepatitis B virus, the H1N1 and H9N2 influenza viruses, and the Herpes simplex virus are all susceptible to the effects of Kaempferol (Zhang et al., 2012; Rahman et al., 2022).

#### 2.1.9. Cholic acid pharmacological activity

Since both Cholic acid and metal ions have pharmacological effects, such as antibacterial, anti-viral, antifungal, antimalarial, antitubercular, anti-cancer, spermicidal, and antiallergic, their organometallic complexes were created to have a synergistic effect (Kishu and Siva, 2011). It has been found that several cholic acid derivatives, including taurolithocholic acid, lithocholic acid 3-sulfate, and taurolithocholic acid 3-sulfate, preferentially suppress type 1 human immunodeficiency virus replication (HIV-1) (Baba et al., 1989). Cholic acid can be prevented Coxsackievirus B3 recurrence and produce anti-viral activity (Han et al., 2018).

#### 2.1.10. Chlorogenic acid pharmacological activity

Chlorogenic acid is a widely distributed natural chemical with numerous significant pharmacological actions, including antioxidant, anti-inflammatory, antibacterial, anti-viral, hypoglycemic, lipid-lowering, anti-cardiovascular, antimutagenic, anti-cancer, and immunomodulatory (Naveed et al., 2018). Chlorogenic acid (CHA) treats viral upper respiratory tract infections brought on by influenza, parainfluenza, and respiratory syncytial viruses (Ding et al., 2017). Besides, another *in silico* investigation has reported that Chlorogenic acid might be effective against SARS-CoV-2 (Aini et al., 2022).

#### 2.1.11. Pinocembrin pharmacological activity

Pinocembrin demonstrated potent antifungal action in a dose-dependent manner against *Penicillium italicum* (Peng et al., 2012). Pinocembrin exhibits anti-viral activity against the reproduction of the Zika virus (Ghildiyal et al., 2020; Huang et al., 2022). Pinocembrin inhibits the Zika virus replication cycle’s post-entry process, and also, it also prevents the formation of viral envelope protein and RNA (Guang and Du, 2006).

#### 2.1.12. Sanguinarine pharmacological activity

Sanguinarine is a plant alkaloid with considerable antiangiogenic, anti-cancer, and anti-viral potential but comparatively moderate toxicity (Feldman et al., 2018). Sanguinarine promotes anti-viral activity by upregulating TLR expression and its downstream mediator in MDM (Tilgner and Shi, 2004). It demonstrated selectivity for MKP-1 over MKP-3, inhibiting cellular MKP-1 with an IC_50_ of 10 m (Guang and Du, 2006).

**Figure 1:**
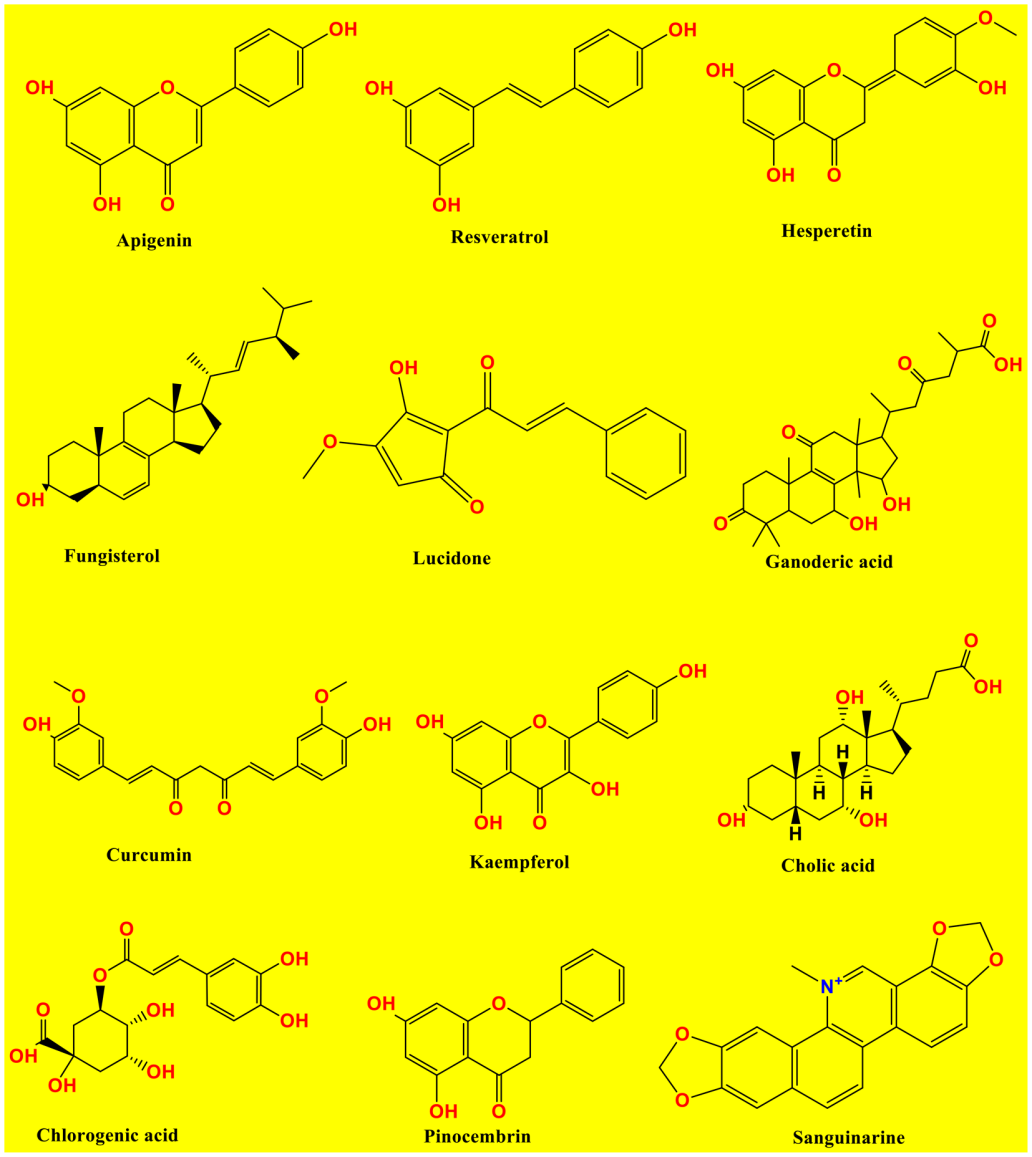
Chemical structures of studied compounds.

Below Figure 1 has shown the chemical structures of studied compounds and Table 1 displayed the pharmacological evidence more precisely.

**Table 1:** Experimental and clinical data on the anti-viral activities of natural compounds against viral strains have been reported in different research investigations.

Drug name	Anti-viral activity	References
Apigenin	Apigenin suppresses enterovirus-71 infection by interfering with RNA-transacting factor interactions	Zhang et al. (2014)
Resveratrol	Resveratrol effectively reduced the cytopathogenic the action of AD virus type 7	Guan et al. (2008)
Hesperetin	Hesperetin inhibits the intracellular reproduction of viruses	Oo et al. (2016)
Fungisterol	Fungisterol’s anti-viral properties related to the polysaccharides present in the mycelium and fruiting bodies	Raut and Adhikari (2021)
Lucidone	Lucidone inhibits DENV protein production	Senthil Kumar and Wang (2016)
Ganoderic acid	Ganoderic acid are effective against human immunodeficiency virus type 1 (HIV-1)	Lindequist et al. (2005)
Curcumin	Curcumin prevented colon cancer cell lines (HT-29 and HCT-15) from proliferating by gathering cells in the G2-M phase	Syng-Ai et al. (2004)
Kaempferol	The 3a channel can be blocked by kaempferol and kaempferol glycosides, providing anti-viral action.	Schwarz et al. (2014)
Cholic acid	Cholic acid suppresses type 1 human immunodeficiency virus replication (HIV-1)	Baba et al. (1989)
Chlorogenic acid	Chlorogenic acid in reducing the HBV DNA relapse	Zuo et al. (2015)
Pinocembrin	Pinocembrin prevents the formation of viral envelope protein and RNA	Guang and Du (2006)
Sanguinarine	Sanguinarine promotes an anti-viral activity by upregulating TLR expression and its downstream mediator in MDM	Sunthamala et al. (2020)

### 2.2. Genome and physiological characteristics

**Figure 2:**
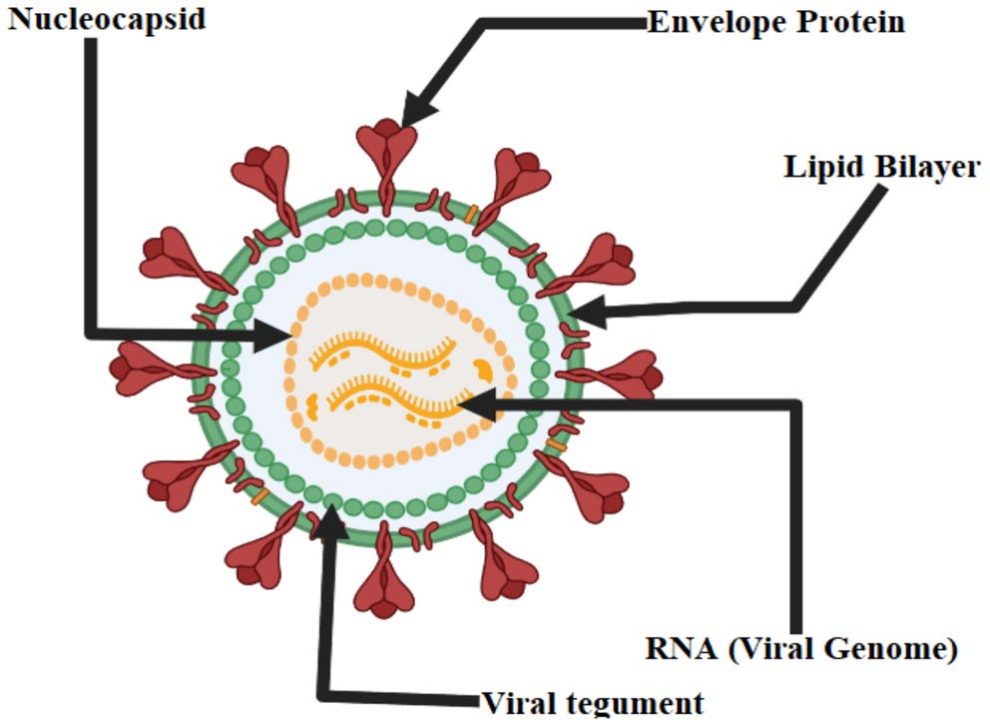
Structure of West Nile Virus.

The genus Flavivirus, of the family Flaviviridae, is host to the West Nile virus. Flavivirus virions are round and have a diameter of around 50 nm. The viral envelope and membrane proteins are encased in a lipid bilayer surrounding the nucleocapsid (roughly 30 nm in diameter and composed of capsid protein and genomic RNA). The virion has a single, plus-sense RNA genome about 11 kilobases in length and is divided into a 5′ untranslated region (UTR). The conserved dinucleotide AG follows the 5′ caps of the flavivirus genome, and the conserved dinucleotide CUOH finishes off the 3′ ends of the genome. The 5′ UTR and 3′ UTR of genomic RNA each have a length of about 100 and 500 to 700 nucleotides, respectively. Viral and cellular proteases co-translate and post-translate the encoded polyprotein into seven nonstructural proteins (NS1, NS2a, NS2b, NS3, NS4a, and NS5) and three structural proteins (capsid, pre-membrane or transmembrane, and envelope). The nonstructural proteins function primarily as replication complex components to ensure the successful replication of viral RNA. For RNA replication to take place, NS1 must interact with NS4a. Hydrophobic NS2a was shown to have a role in flavivirus particle formation and release. With NS3, NS2b generated a complex essential for NS3’s serine protease activity. A serine protease, 5′-RNA triphosphatase, nucleoside triphosphatase, and RNA helicase are all functions of NS3, a multifunctional protein. Yet, the roles of NS4a and NS4b, two membrane-associated proteins, remain unknown. The NS5 protein is an RNA-dependent RNA polymerase and a methyltransferase that modifies the 5’ RNA cap structure. When a flavivirus replicates, a replication complex is assembled on the 3′ UTR of the genomic RNA during the process of co-translation. When the positive-sense RNA is copied, the resulting negative-sense RNA is still base-paired with the original replicative form (RF). After that, the RF is reused as a starting point for the asymmetric production of more positive-sense RNA (Figure 2) (Tilgner and Shi, 2004).

### 2.3. Transmission and clinical manifestation

**Figure 3:**
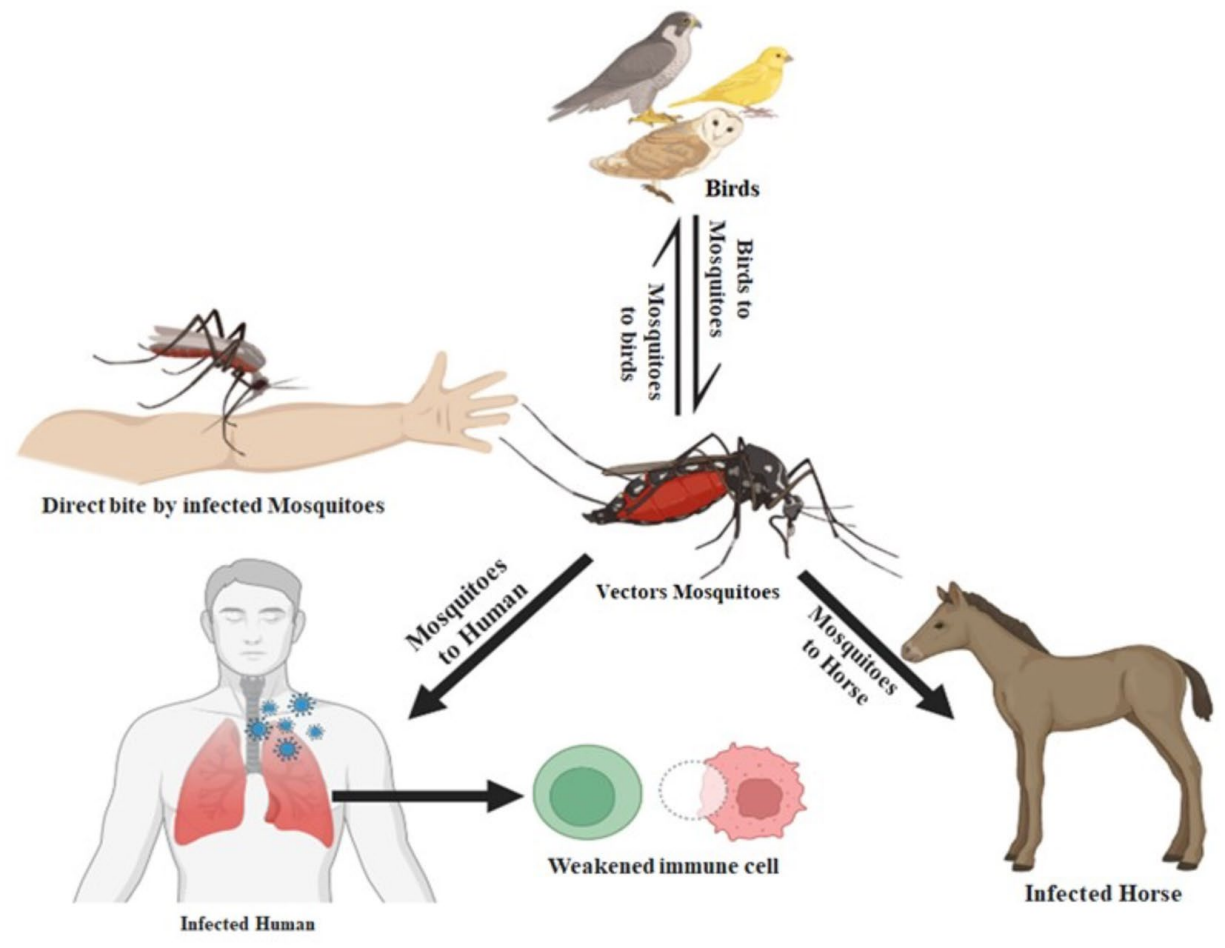
The West Nile virus transmission from animals to humans. When an infected mosquito bites an animal or a person (the “host”), the virus is introduced into the host’s circulation and has the potential to severely sick the host. The virus enters the circulation of the mosquito when an infected bird bites it and ultimately travels to its salivary glands.

The West Nile virus infection is a mosquito-borne disease that may infect people, birds, and animals. According to the CDC, the primary host for the virus is birds, and mosquitoes acquire the virus by biting affected birds. Health experts have announced that it cannot be transmitted from direct contact from person to person (West Nile Virus Found in Another NY County, Bringing Case Total to 5, n.d.). Figure 3 displays the transmission routes of the West Nile virus.

## 3. Computational method and material

### 3.1. Preparation of ligand and geometry optimization

**Figure 4:**
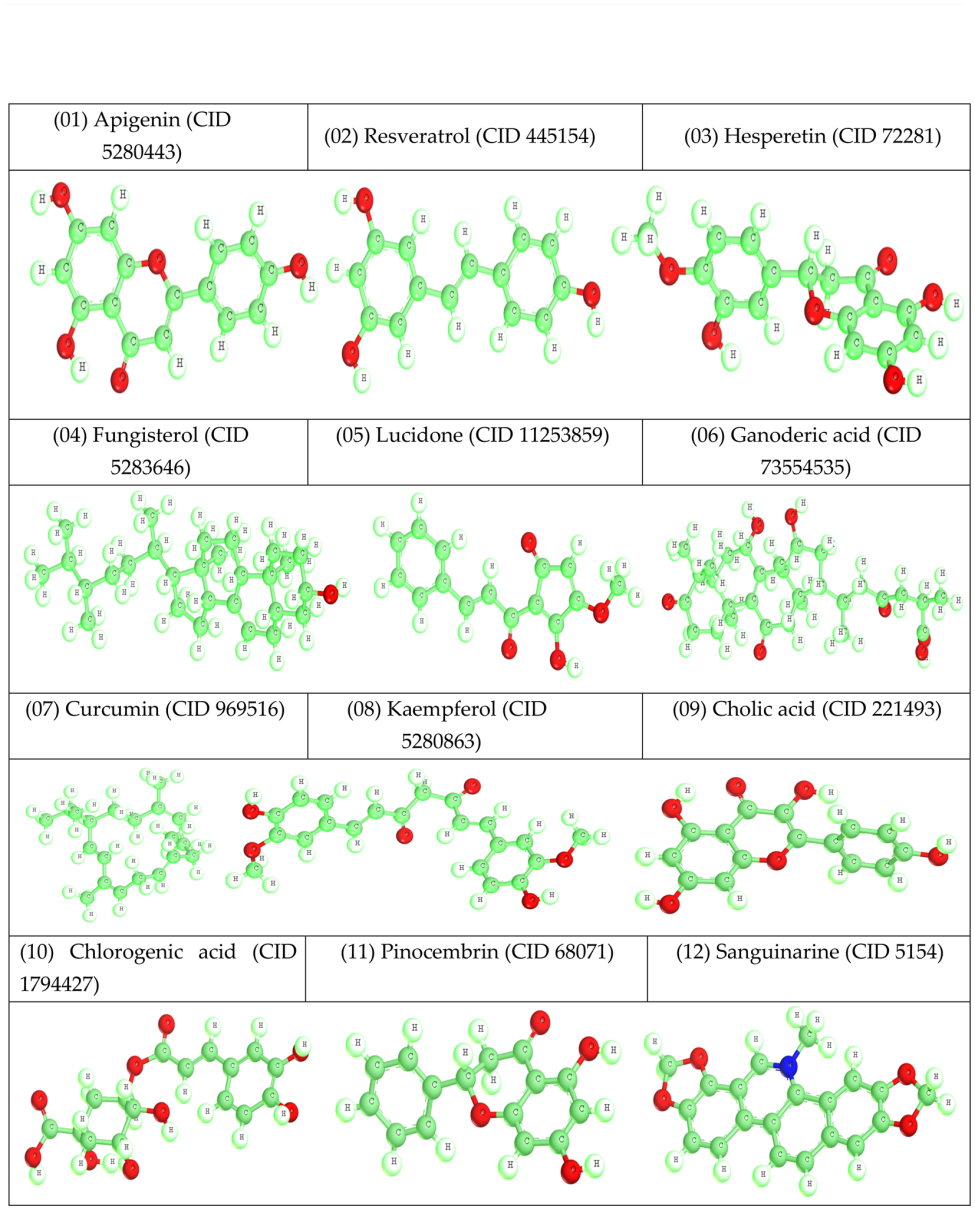
Optimized structure of reported natural compounds.

By utilizing vibrational frequency from the DMol3 code of Material Studio 08, a method known as DFT functional was used to accomplish molecular optimization (Yuan et al., 2016; Kumer et al., 2021). The molecular optimization was performed to produce exceptionally accurate results during molecular docking. After optimizing, chemical compounds were generated as a pdb file for molecular docking, drug-likeness, and ADMET (Figure 4).

### 3.2. Determination of ADMET, Lipinski rule, and drug-likeness

To assess their drug likeliness, we examine the ADMET (Absorption, Distribution, Metabolism, Excretion, Toxicity) pharmacological characteristics of chosen compounds (Wijaya et al., 2021). With the aid of ADMET, we may predict if a molecule will be successfully implemented while designing new drugs based on their pharmacokinetics qualities. The pkCSM web server and Swiss-ADME were employed for this (AI Azzam, 2023). The Lipinski rule violation, GI (gastrointestinal) absorption, BBB penetration, and solubility are some of the pharmacological issues discussed by the Swiss-ADME online-based web server.^1^ We research our compound’s excretion and toxicity using the pkCSM web server (Daina et al., 2017; Wijaya et al., 2021).^2^ Drug likeness is one of a drug compound’s sensory attributes that is frequently used in the drug discovery process. When Lipinski and colleagues released the rule of 5 (Ro5) in 1997, it was based on research of 2,245 drug qualities from the World Drug Index (WDI) databank that had been accepted for phase 2 clinical trials (Sadowski and Kubinyi, 1998; Akash, 2022). This was the first time that pharmacokinetic properties had been approached academically. Molecular weight, the amount of hydrogen bond donors and acceptors, topological polar surface area, consensus log Po/w, and drug similarity features such as lipinski rule violation and synthesis accessibility is all examined in this section.

### 3.3. Protein preparation, molecular docking analysis

**Figure 5:**
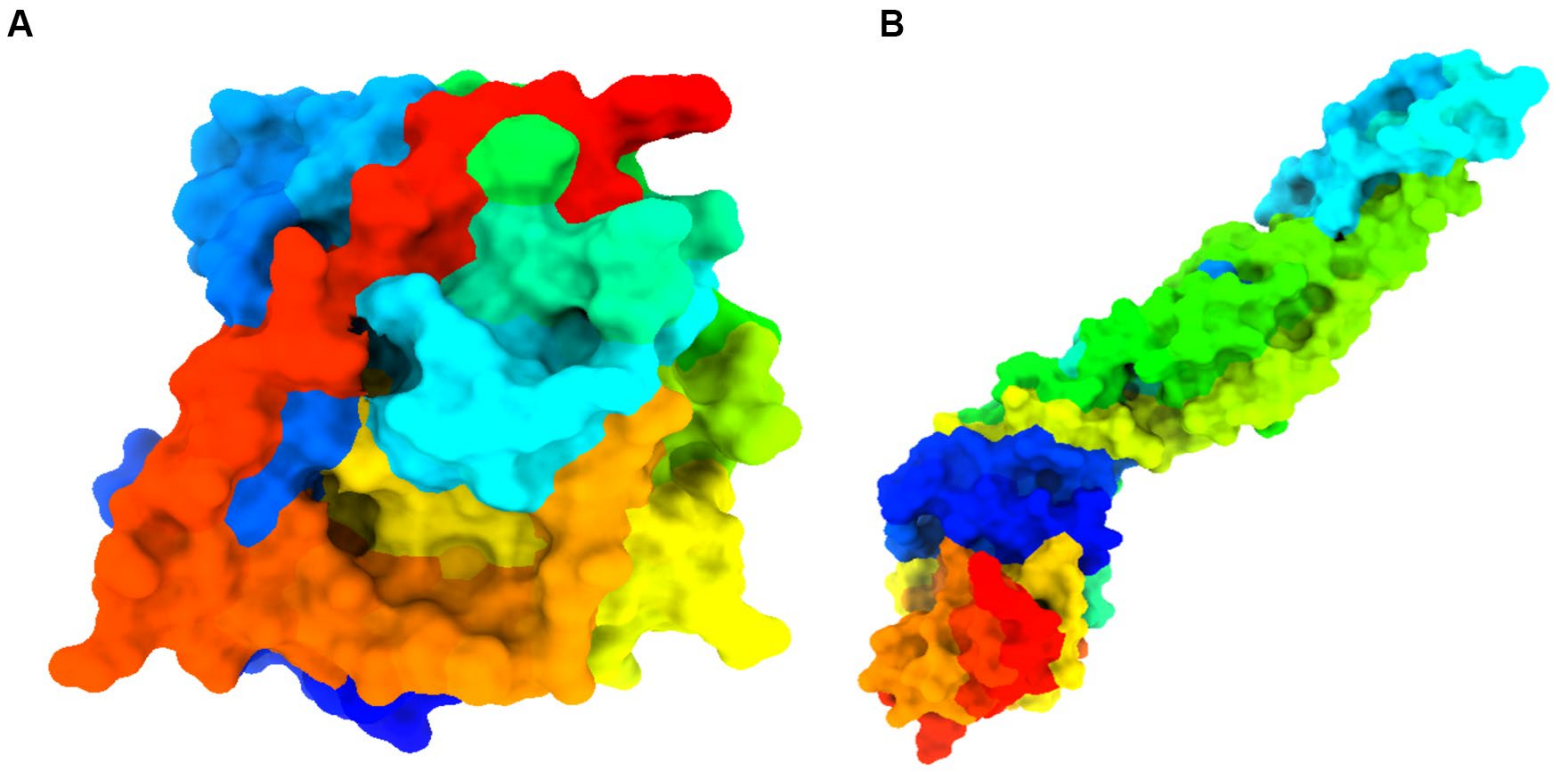
Details information about studied proteins. **(A)** West Nile virus methyltransferase (PDB ID 2OY0). Organism: West Nile virus. Method: X-ray diffraction. Resolution: 2.80 (Zhou et al., 2007). **(B)** West Nile virus envelope glycoprotein (PDB ID 2I69). Organism: West Nile virus. Method: X-ray diffraction. Resolution: 3.11 (Kanai et al., 2006).

The crystal structure of several west Nile virus strains was retrieved from the Protein Data Bank (PDB) of the RCSB Protein online portal (Burley et al., 2017). Protein purification was carried out carefully by removing ligands and water using Pymol v2.4.1 software^3^ (Padmi et al., 2022) (illustrated in Figure 5). The crystal structure of the cleaned protein file is fed into AutoDock tools after the extra water and ligand have been removed, and the instructions are followed to create the pdbqt file format (Yuan et al., 2017; Kumer et al., 2022).

The development of the binding affinity of molecular complex may be predicted *via* the use of a computer approach known as “*in silico* molecular docking” (Agarwal and Mehrotra, 2016). In current studies, the PyRx AutoDock vina tool is applied for molecular docking. Before, performing docking, previously prepared drug, and protein were uploaded in PyRx application, and converted them as autodocking protein, and autodocking protein. Finally, specified grid parameter were selected, and perform the molecular docking studies (Yuliana et al., 2013).

### 3.4. Molecular dynamic simulations (MDs) methods

MD modeling is used to study the movement of molecules and atoms in dynamic systems, like protein-ligand complexes, in order to understand important physicochemical phenomena. MD simulations were run using receptor-ligand complexes acquired *via* molecular docking research to verify the binding mechanism and stability in a dynamic system. For this purpose, three best-docked compounds were subjected to 100 ns MD simulations: apigenin and curcumin against the West Nile virus methyltransferase with PDB ID 2OY0 as the target receptor, and Ganoderic acid against the West Nile virus envelope glycoprotein (PDB ID 2I69) target receptor. The simulation system was set up using the CHARMM-GUI web-based graphical interface and produced the force field for both the ligands and the proteins (Wang et al., 2019). In the simulations that were carried out for 100 ns inside a periodic water box, the CHARMM36 force field and the Gromacs version 2020 software package were both utilized (Akash et al., 2022; Terefe and Ghosh, 2022). The complexes were positioned within a rectangular box that had a buffer distance of 10 in each of the cardinal directions. The box was subsequently dissolved in water molecules containing TIP3P. To keep the systems neutral, sodium and chloride ions were introduced, and then the energy was minimized using the steepest descent approach. Equilibration was performed on each of the complete systems at a temperature of 310 K for a total of 5,000 steps (i.e., 10 PS). The NPT ensemble’s performance lasted 100 s. The Lincs technique was used to place constraints on hydrogen, and as a result, the timestep was set at 2 fs. All van der Waals forces were calculated using a switching method between 12 and 14, and 14 was found to be the cutoff value. The particle mesh Ewald (PME) method with a maximum grid spacing of 1.2 was used to figure out the long-range electrostatic interactions. No multiple-time-stepping strategy was used; instead, PME computations were performed at each step. At a temperature of 310 kelvin, the initial velocities were chosen at random from a Maxwellian distribution. The temperature was maintained at 310 K. The barostat was programmed to have a target of 1 bar for any system size changes that occurred. The integration time step was 2 fs. After then, the simulation’s output was re-centered, and the trajectories were subsequently assessed using the VMD (University of Illinois at Urbana-Champaign, Urbana, IL, USA) program, Bio3D, and QTGRACE, respectively. In the investigation of the system’s stability, the root means square deviation (RMSD), the root means square fluctuation (RMSF), the radius of gyration (Rg), the number of hydrogen bonds, and the principal component analysis were taken into consideration (Walters, 2012).

### 3.5. Binding free energy calculation using MM-PBSA

In the molecular dynamics simulation, free energy calculation takes a major role in determining the binding energy of ligands inside proteins (Zikri et al., 2020). In this investigation, the MM-PBSA method was used to calculate the free interaction energy between ligands and West Nile virus methyltransferase (PDB ID 2OY0) and West Nile virus envelope glycoprotein (PDB ID 2I69). Calculations using MM-PBSA were performed to determine the binding affinity of protein-ligand complexes. Binding free energy (ΔG) estimation was done by eq. (3) using the script MMPBSA.py of the AMBER package (Wang et al., 2019).


(1) [ΔGbindG=−complexG−proteinG−ligand]

G-complex is the free energy of the complex; G-receptor is the free energy of the receptor; G-ligand is the free energy of the ligand (Terefe and Ghosh, 2022).

## 4. Result and analysis

### 4.1. Lipinski rule, pharmacokinetics

The Lipinski rule and drug resemblance give important details about the initial stages of medication development and raise the probability of success. According to the Lipinski rule of five, the drug’s permeability and bioavailability depend on the molecular weight, the number of hydrogen bond donors and acceptors, and Topological polar surface area (Å^2^). The six-ligand compound can successfully pass the Lipinski rule of five without any violations (Walters, 2012; Akash et al., 2022). However, the other six ligand compound cannot satisfy by Lipinski rule. Besides, low-molecular-weight ligand compounds are more readily absorbed, dispersed, and transported than those with greater molecular weights (Santos et al., 2016). The polarity of a ligand compound can be determined by a molecule’s topological polar surface area (TPSA), which is a significant pharmacokinetics feature. These values help describe the drug transport characteristics. All polar atoms, primarily oxygen and nitrogen with connected hydrogen, make up the polar surface area of molecules. All our ligands showed very promising pharmacokinetics properties shown in the Table 2.

**Table 2:** Predicted data of Lipinski rule, pharmacokinetics.

No.	Weight (g/mol)	Hydrogen bond acceptor	Hydrogen bond donor	Topological polar surface area (Å^2^)	Lipinski rule	
					Result	Violations
01	270.24	05	03	90.90	Yes	00
02	228.24	03	03	60.69	No	01
03	302.28	06	03	96.22	Yes	00
04	400.68	01	01	20.23	No	02
05	70.18	04	01	63.60	Yes	00
06	368.38	06	02	93.06	No	02
07	368.38	06	02	93.06	No	02
08	286.24	06	04	111.13	Yes	00
09	408.57	05	04	97.99	No	01
10	354.31	09	06	164.75	Yes	01
11	256.25	04	02	66.76	Yes	00
12	332.33	04	00	40.80	No	01

### 4.2. Molecular docking analysis against West Nile virus

One of the core technologies in computer-aided drug design is molecular docking, which allows for the analysis of protein-ligand complexes’ non-bond interactions and energy bindings. Several natural compound derivatives were studied to attach molecularly with the target receptor protein of the West Nile virus. Molecular docking has been used to obtain specific information on protein-ligand complexes’ interaction and binding affinity. The compounds were evaluated by utilizing the interaction with the maximum binding affinity scores. The use of computer-based docking studies in drug design is essential because it enables examination of the ligand configuration with the receptor protein’s active site and investigation of non-bond interaction. Typically, it was believed that a pharmaceutical drug’s effective binding affinity was −6.00 kcal/mol is expected (Kawsar et al., 2022; Rahman et al., 2022). The most incredible binding energy for the West Nile virus methyltransferase with (PDB ID: 2OY0) target receptor is −8.3 kcal/mole, whereas the maximum binding energy for the West Nile virus envelope glycoprotein with (PDB ID: 2I69) target receptor is −8.1 kcal/mol, as observed in molecules 04 and 20 of our medication (Table 3). That suggests our pharmacological compound is working more effectively against these two targets. As a result, our therapeutic molecule may be particularly successful in treating West Nile virus infection.

**Table 3:** Summary of binding affinities.

No.	Name	West Nile virus methyltransferase (PDB ID 2OY0)	West Nile virus envelope glycoprotein (PDB ID 2I69)
		Binding Affinity (kcal/mol)	Binding Affinity(kcal/mol)
01	Apigenin	−8.5	−7.5
02	Resveratrol	−7.7	−7.2
03	Hesperetin	−8.0	−7.1
04	Fungisterol	−7.4	−7.0
05	Lucidone	−7.8	−7.4
06	Ganoderic acid	−7.6	−8.0
07	Curcumin	−8.3	−7.3
08	Kaempferol	−8.1	−6.8
09	Cholic acid	−7.9	−7.6
10	Chlorogenic acid	−8.0	−7.1
11	Pinocembrin	−7.5	−6.5
12	Sanguinarine	−7.2	−6.7

### 4.3. Protein-ligand interaction valuation

Docking studies of West Nile virus methyltransferase (PDB ID 2OY0) have revealed that drugs like Apigenin, and Curcumin, had the greatest binding score. On the other hand, compounds such as Ganoderic acid, and Cholic acid have been found to dock with the West Nile virus envelope glycoprotein (PDB ID: 2I69) with the highest binding score. So, the protein-ligand interaction valuation of these mentioned compounds was carried out by BIOVIA Discovery Studio 2016 v16.1.0 and Pymol v2.4.1 software (Wahyuni et al., 2022). Curcumin’s binding sites are located at LYS-A:105 and ILE-A:147 on West Nile virus methyltransferase (PDB ID 2OY0), Apigenin binding site are found at VAL-A:132, ASP-A:131, PHE-A:133, LYS-A:105, ASP-A:146, TRP-A:87, GLY-A:58, ASP-A: 79. On the other hand, Ganoderic acid binding sites are located at PRO-A:339, VAL-A:304, LYS-A:337, ARG-A:338, VAL-A:385, VAL-A:343, ASA-A:347. Cholic acid binding sites are located at LE-A:340, VAL-A:358, VAL-A:304, LYS-A:337, SER-A:341, and PRO-A:339. Sanguinarine binding sites are located at ALA-A:161, PRO-A:146, SER-A:363, VAL-A:364, ALA-A:369, LYS-A:370, and THR-A:148. This active side was predicted by discovery studio 2021 (Kumer et al., 2022).

**Figure 6:**
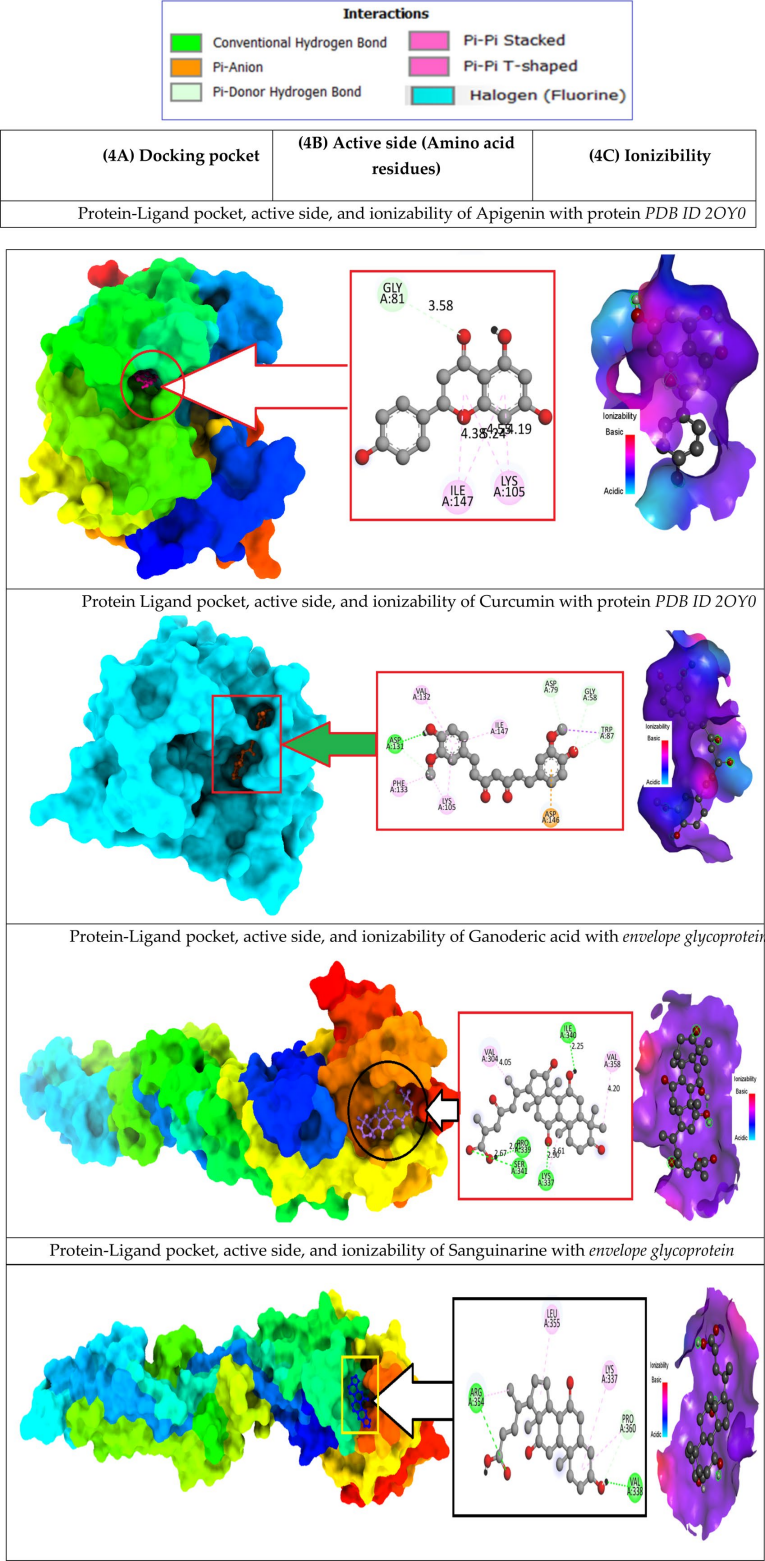
Docking pocket, active side (Amino acid residues), and ionizability.

Besides, Un-ionized medications are administration into the body more effectively than their ionized counterparts, which are also known as charged drugs. A drug that is a weak acid will be absorbed predominantly in the acidic medium if it is administered orally; on the other hand, a drug that is a weak base will be captured in the alkaline system of the small intestines if it is administered orally (Song et al., 2004; Kumar et al., 2013). In (6C, as depicted in Figure 6) ionizability is displayed. The red color represented basicity, and sky-blue represented acidic condition while the deep blue color means neutral condition. According to the study, it is seen that most of the drugs are slightly basic in nature, which means they may better absorption rate in basic medium.

### 4.4. MD simulation results

The molecular dynamics simulation was launched to comprehend the stability of the complex in depth. This approach is essential for understanding the structure–function relationship of macromolecules. In this study, Apigenin, Curcumin, and Ganoderic Acid were determined to be the best leaders among all the natural compounds evaluated due to their consistent performance in terms of docking score and protein-ligand interactions. To validate this further, the docked complexes of selected compounds, i.e., apigenin and curcumin with West Nile virus methyltransferase (PDB ID 2OY0) and Ganoderic acid with West Nile virus envelope glycoprotein (PDB ID 2I69), were studied for the complexes’ stability and intermolecular interaction with respect to time by 100 ns molecular dynamics (MD) simulation. When simulations were completed, the root mean square deviation (RMSD), root mean square fluctuation (RMSF), the radius of gyration (Rg), H-bond analysis, and principal component analysis (PCA) were determined for each frame of the trajectory.

#### 4.4.1. Study of stability using root mean square deviation

**Figure 7:**
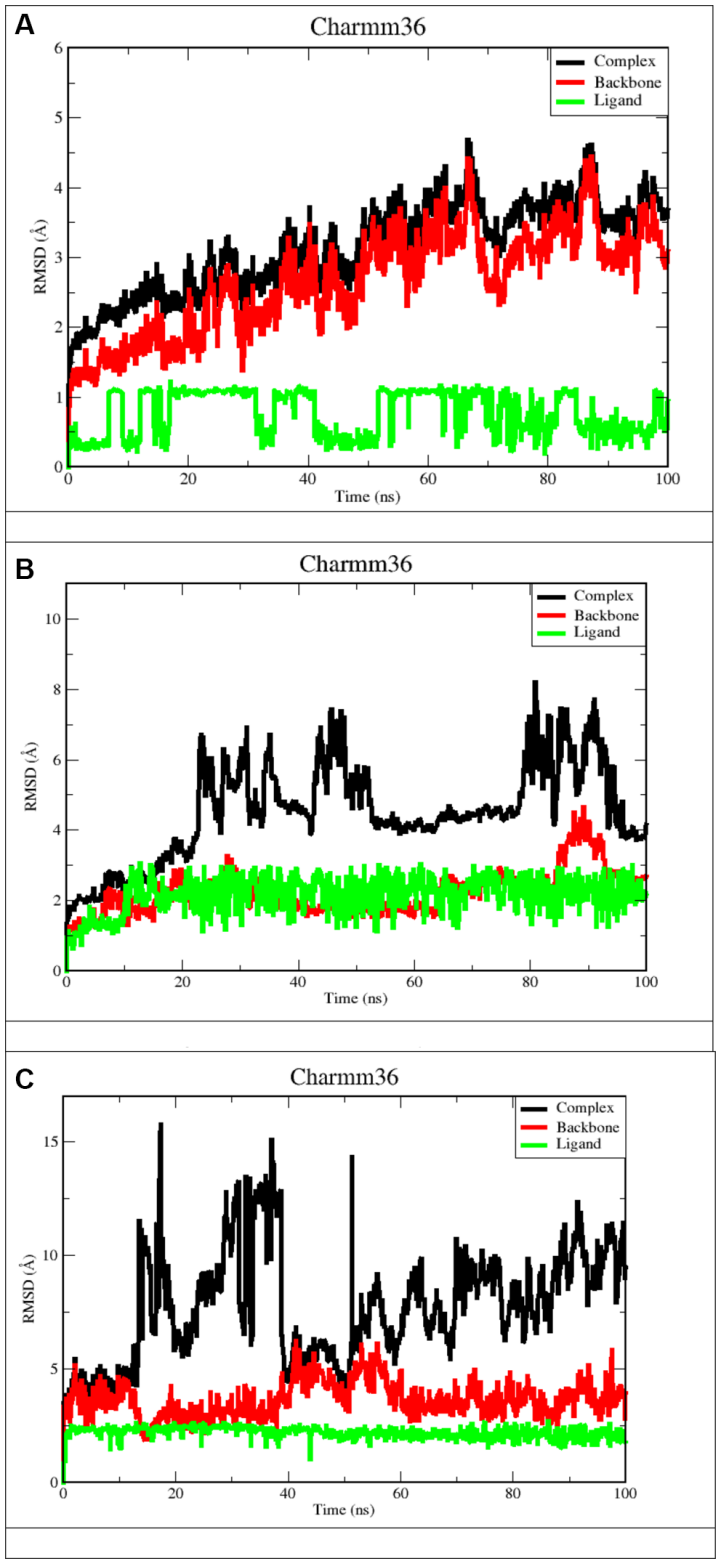
RMSD plots after 100 ns run. **(A)** *Apigenin* against West Nile virus methyltransferase (PDB ID 2OY0). **(B)** *Curcumin* against West Nile virus methyltransferase (PDB ID 2OY0). **(C)** *Ganoderic acid* against West Nile virus envelope glycoprotein (PDB ID 2169).

RMSD refers to the occurrence of deviations that were noticed during the evolution of the simulation (Peng et al., 2012; Ghildiyal et al., 2020; Huang et al., 2022). Furthermore, the RMSD defines the stability of the structural as the smaller the RMSD, the greater the stability (Huang et al., 2022). RMSD calculations for protein backbones, ligands, and complexes during a100 ns MD simulation of each protein-ligand complex were used to get an insight into conformational changes during protein-ligand interactions, as shown in Figure 7.

Derived from a 100 ns MD simulation, the RMSD of the ligand, protein backbone, and complex RMSD values for apigenin, curcumin, and Ganoderic acid are displayed. The RMSD of the protein backbone is displayed in red. The complex RMSD (root-mean-square deviation) is depicted in black. The RMSD (root mean square deviation) of the ligand is depicted in green.

Figures 7A,B illustrate that the West Nile virus methyltransferase protein with apigenin ligand has a lower RMSD value compared to the West Nile virus methyltransferase protein with curcumin ligand. In Figures 7A,B, Ligand’s average RMSD is 0.6785 Å and 2.066 Å, respectively. Although the apigenin ligand bound the West Nile virus methyltransferase protein, the low RMSD value showed large fluctuations throughout the simulation. In the case of the ligand curcumin, the fluctuation was just observed during the first 10 ns of trajectory. Furthermore, the ligand RMSD stabilized again after 10 ns and remained consistent till the end of the simulation time of 100 ns. This indicates that the curcumin ligand contacts remained intact during the simulation. According to Figure 7C, the Ganoderic acid ligand-bound West Nile virus envelope glycoprotein showed a stable RMSD value during the simulation time. The trajectories were analyzed with the VMD program, and the results showed that the ligands curcumin and Ganoderic acid did not jump out of the domain of the protein, indicating that they were located within the binding site. This was discovered when we found that neither of the ligands jumped out of the domain. According to the results of the root-mean-square-difference (RMSD) analysis of the ligands, neither the binding orientation of the curcumin nor the Ganoderic acid ligands changed during the simulation. Apigenin and Ganoderic acid have maintained the same conformational orientation throughout their whole structures and have not deviated from them. This suggests that, in the case of the West Nile virus methyltransferase protein-bound Apigenin-ligand, numerous binding orientations were observed and that this ligand shifted positions throughout MD simulations and departed the binding pocket. The docking position was deemed unacceptable for the candidate with a complex structure. During the 100 ns simulation, the RMSD analysis of backbone atoms for the two proteins, West Nile virus methyltransferase and West Nile virus envelope glycoprotein, revealed different conformational states in terms of the protein’s backbone. Indeed, curcumin and Ganoderic acid tend to reach a constant equilibrium, but the RMSD of the apigenin backbone was considerably high. The backbone of Apigenin remained distinct throughout the simulation, resulting in a maximum RMSD of 4.5 Å. This difference in the deviation range explains the change in stability of West Nile virus methyltransferase, which represents the effect of the substituted amino acid on the structure of the protein. The RMSD plots of Apigenin and Curcumin’s backbones have average values of 2.91 and 1.76, respectively. The first 30 ns of the simulation of curcumin showed instability of the protein, but from 30 to 60 ns of simulation, the protein was stable. The first stable conformation of the Ganoderic acid backbone occurs between 20 and 40 nanoseconds, while the second conformation occurs between 60 and 100 nanoseconds. The RMSD remains constant at 1.7 Å, and between 20 ns and 40 ns, RMSD >5 Å is observed to fluctuate significantly. Among the three trajectories, the apigenin backbone protein exhibited the greatest variation and the highest RMSD value. As opposed to Apigenin and Curcumin, the Ganoderic acid protein backbone exhibited less divergent patterns. In addition, according to Figure 7, the average RMSD values for the three complexes were 3.50 Å, 3.97 Å, and 5.50 Å, respectively. As determined by RMSDs, the protein-ligand complex in Figure 7A exhibited a rising fluctuation, whereas in Figure 7B, the complex is equilibrated between 50 and 75 ns. Also, as can be seen in the complicated Figure 7C, the trajectory is initially in equilibrium for 5 ns, then has a big increase to 16 at 20 ns, and finally experiences a substantial reduction to 5.3 at roughly 23 ns. From then on, the complex exhibits a wide range of fluctuations, from nearly stable to extremely unstable, until it reaches 70 ns. In the last 30 ns, the complex has shown persistent, minute fluctuations. As a result, the West Nile virus methyltransferase complex protein-bound apigenin exhibited the lowest RMSD value compared to the curcumin compound and Ganoderic acid compound complexes; however, this did not prove its greater stability and fewer conformational changes compared to other complexes. At the conclusion of the simulation, the ligand appeared highly unstable when complexed with apigenin. The apigenin ligand exhibited substantial variations, indicating the general instability of the complex.

#### 4.4.2. Root mean square fluctuations

**Figure 8:**
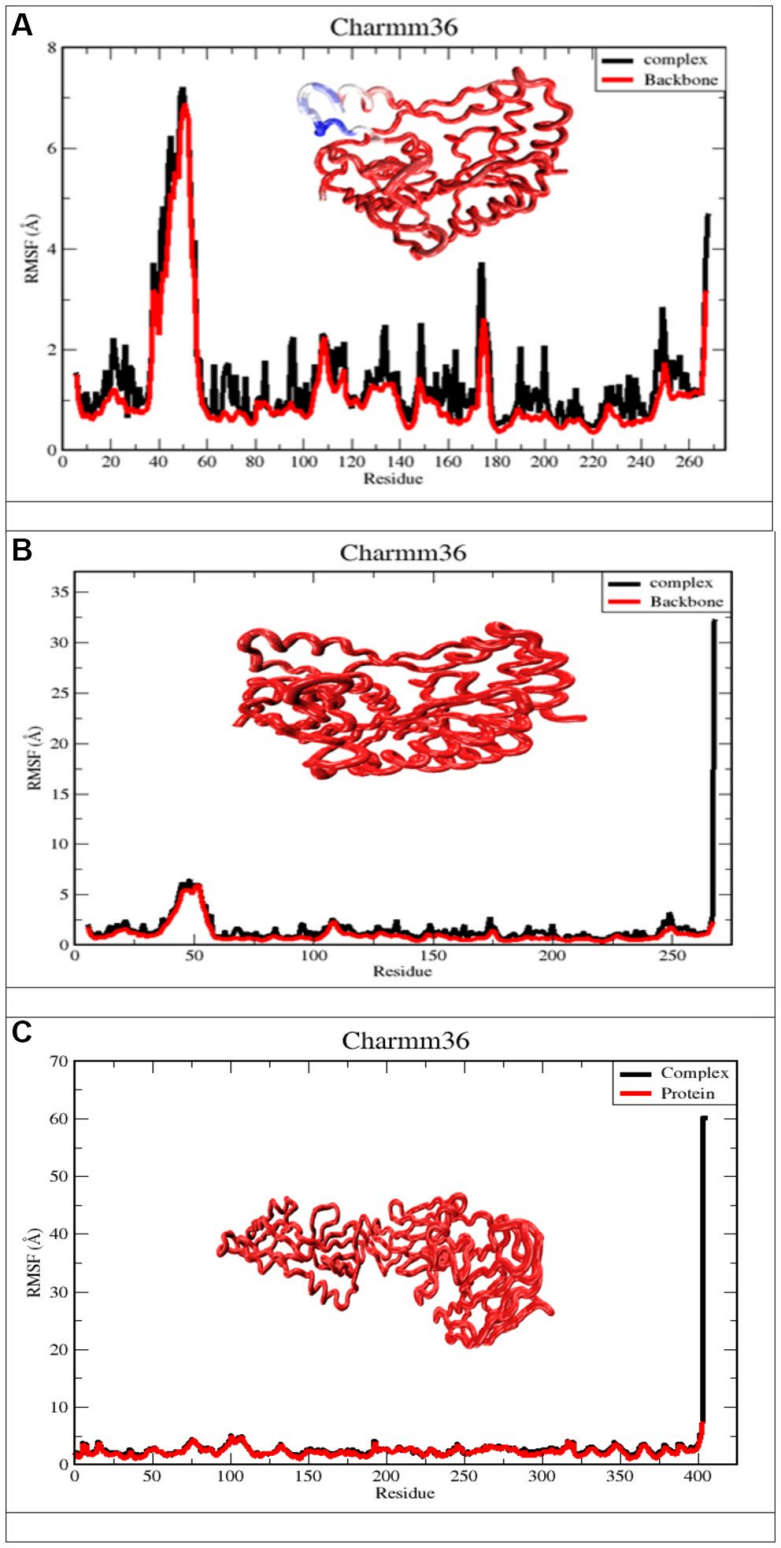
Derived from a 100 ns MD simulation, the RMSF of a complicated protein backbone. The RMSF of the protein backbone is depicted in red. The complex’s RMSF is depicted in black. **(A)** *Apigenin* against West Nile virus methyltransferase (PDB ID 2OY0). **(B)** *Curcumin* against West Nile virus methyltransferase (PDB ID 2OY0). **(C)** *Ganoderic acid* against West Nile virus envelope glycoprotein (PDB ID 2169).

RMSF measures the amount by which atomic locations have deviated from their initial positions and illustrates the dynamic nature of the protein-ligand interaction. In other words, RMSF exemplifies how dynamic the interaction between the protein and the ligand is. As a result, the RMSF data of the protein backbone and complexes were plotted to visualize the average fluctuation of all the amino acid residues, as depicted in Figure 8 for a 100 ns MD trajectory. Furthermore, the RMSF value can be used to assess the significance of individual protein residues in preserving the native shape of a protein-ligand complex. A high RMSF number indicates greater flexibility, while a low RMSF value indicates a more stable zone. Hence, a higher number of RMSF residues or groups suggests a higher degree of flexibility, which in turn suggests a higher probability of interaction with ligand molecules. Moreover, reduced RMSF fluctuations are associated with lesser flexibility, resulting in diminished interaction potential. Figure 8A depicts the RMSF analysis for the ligand apigenin. None of the nine residues with a high RMSF value (RMSF value >3) were outside of protein binding sites. In addition, fluctuations of substantial peaks in the RMSF graph were found at LYS45, GLU46, GLY47, ASN48, VAL49, THR50, GLY51, GLY52, and HSD53, which are shown in blue on the protein. Intriguingly, these residues are not part of the binding pocket, and it is hypothesized that they have no significant effect on the ligand-binding process. The curcumin ligand has four different residues that have high RMSF, but none of them are in the protein binding sites. The Ganoderic acid ligand has not exhibited significant fluctuations with low RMSF values, none of which are in protein-binding sites. As demonstrated in Figure 8A, the RMSF values of the apigenin ligand complex are greater than those of the curcumin structure. The high RMSF values of apigenin indicated a greater degree of flexibility and instability in the protein, whereas the low RMSF values of the curcumin and Ganoderic acid complexes indicated restricted movement of the residues and a rigid structure in the presence of the ligands for West Nile virus methyltransferase. According to the information presented above, the findings of curcumin and Ganoderic acid have demonstrated that, in general, the protein was stable and did not undergo any significant changes. In this investigation, apigenin and protein interactions with RMSF values greater than 2.5 are regarded as having fewer stable bonds. Figure 8, on the other hand, reveals a negligible distinction between the fluctuation scores of the residues in the protein-curcumin combination and the protein-Ganoderic acid complex. In Figures 8B,C, the RMSF plot of ligand-protein for each ligand reveals that the RMSF value at the C-terminal residues (end) is quite high because these residues represent the tails or ends of the protein structure, which are highly reactive and free to move. Each of the two complex system residues, VAL267 (C-terminal), exhibits significantly higher fluctuations than other residues. This is likely because it is placed at the end of the protein chain, allowing it to be more flexible than other residues. In Figure 8C, the C-terminal residue changed significantly, with RMSF values of 60, whereas for curcumin, it reached 30. Figure 8C depicts the creation of a highly mobile free-end loop in Ganoderic acid, which may account for the significant variation observed.

#### 4.4.3. Radius of gyration analysis

**Figure 9:**
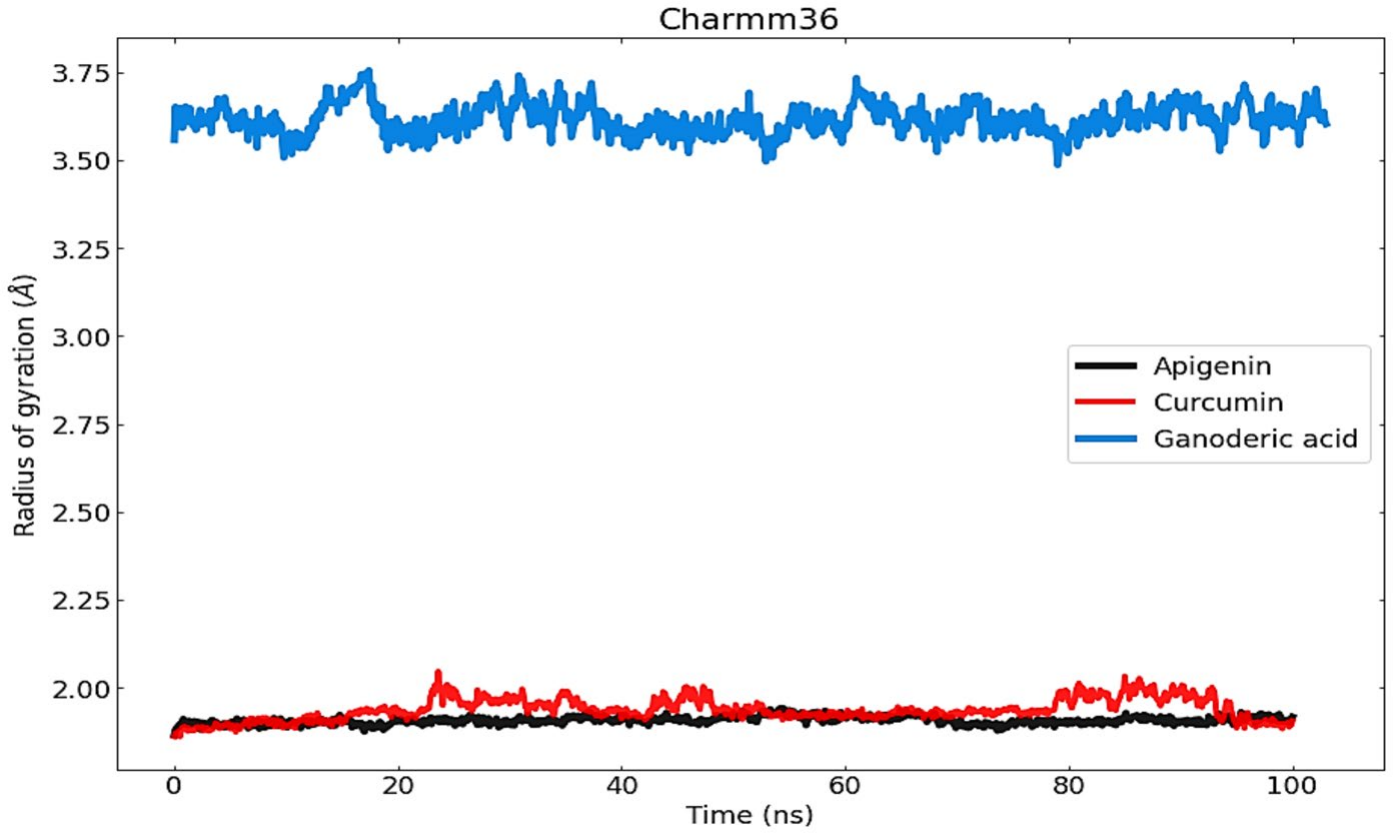
The radius of gyration (Rg) was used to measure and comprehend the compactness of protein complexes and their structure.

To evaluate structural compression changes, the gyration radius diagram of each structure was recorded over the course of the simulation. To determine the compactness of the system over time, Rg was calculated, with higher Rg values indicating less compactness (more unfolded) with conformational entropy and lower Rg values indicating high compactness with greater structural stability (more folded). Figure 9 demonstrates that the simulation Rg values for the three complexes are 1 Å, 9 Å, 1 Å, 975 Å and 3.5 Å, respectively. From the Apigenin plot, it can be seen that the system exhibited little change until 100 ns, with the exception of a few unfolding events between 40 and 70 ns, after which it stabilized until the end of the simulation. The stability of the protein in the complex was demonstrated by the Rg value, which showed less variation as a result of the fewer changes it experienced. Curcumin, on the other hand, exhibits only very modest fluctuations at the beginning but afterwards demonstrates a significant increase that reaches a Rg value of 2.5 Å between 20 and 40 ns. In the case of Ganoderic acid, the structure showed a sharp increase of Rg up to 3.75 Å at 10 ns, then an immediate decrease of the Rg value towards 3.50 Å, then an increase towards 3.70 Å between 20 ns and 40 ns, then a gradual fluctuation decreases of Rg towards 3.60 Å until the 80 ns. At 80 ns, there is a sharp decrease in the Rg value towards 3.50 Å. After that, an average Rg value of 3.60 A was equilibrated to small fluctuations until the simulation ended. The RG findings demonstrated that the binding of these three molecules induces structural modifications. In general, the patterns of change in RG values across all complexes were distinct. According to the results, the Apigenin complex had the smallest Rg value, which may indicate that it is more compact than the other complexes. A loss of compactness may occur as a consequence of a change in the interaction pattern between the protein and the ligand. The dissolution of hydrogen bonds between molecules might possibly be explained by the curcumin complex’s elevated Rg value. It is also possible that conformational changes in the protein structure were induced by the interaction of curcumin with proteins, which dramatically altered the curcumin complex’s microenvironment. In addition, the Ganoderic acid complex has been reported to have experienced compositional variations, which indicates a less tightly bound structure.

#### 4.4.4. Hydrogen bond analysis

**Figure 10:**
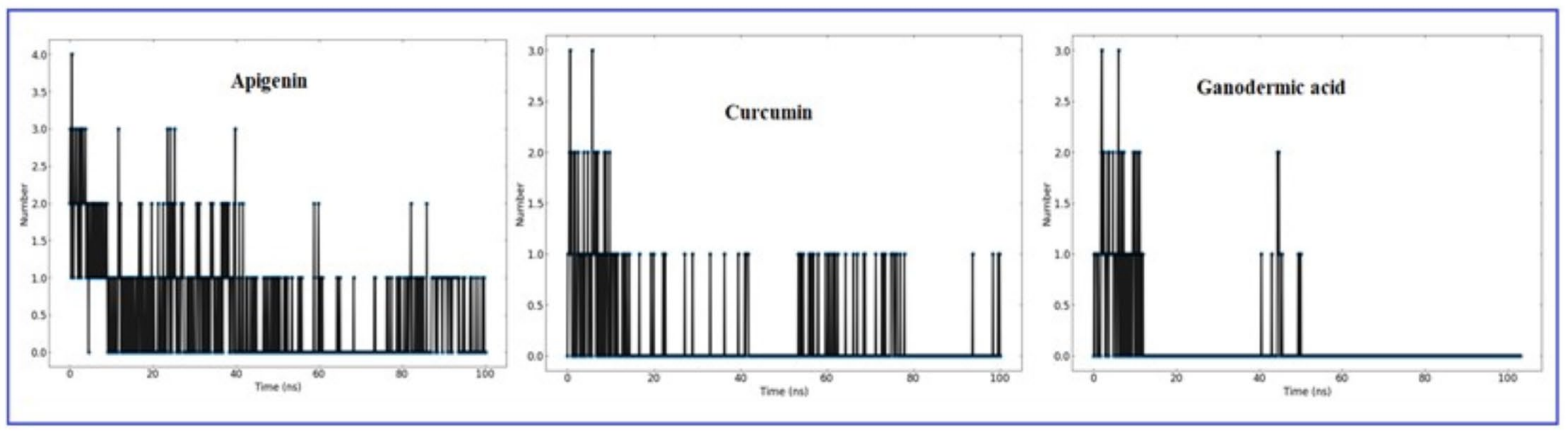
The number of H-bonds formed by the ligand molecule, with the proteins (Apigenin, Curcumin and Ganoderic acid) was obtained from 100 ns MD simulation.

It is worthwhile to investigate hydrogen bonding interactions because they contribute to the binding process and, in particular, persistent hydrogen bonds. Using the VMD hydrogen bond analysis tool, all conceivable hydrogen bonding interactions between the two specified areas, in this case the protein and the ligand, have been explored throughout time. Hydrogen bonds are recognized, and the output contains the overall number of hydrogen bonds as well as their occupancy over time. The ‘Percentage occupancy of the Hbond’ output of the hydrogen bond analysis tool provides access to these values (Figure 10). In docked complexes, significantly more stable hydrogen bonds are reported to be established. During the Molecular Dynamics (MD) simulations, the H-bonds that were present in the docking structures were not only preserved, but additional H-bonds were also found. Apigenin, curcumin, and Ganoderic acid each have their own individual occupancies of identified H-bonds, which are listed in Table 4.

**Table 4:** Analysis of H-bond occupancies for each ligand during MD simulation.

	Donor acceptor	Occupancy
Apigenin	Apigenin-Side-O3 GLY81-Main-O	0.90%
	Apigenin-Side-O3 ILE147- Main-O	0.10%
	LYS105-Main-N Apigenin-Side-O2	11.28%
	LYS105-Main-N Apigenin-Side-O5	3.19%
	LYS105-Side-N2 Apigenin-Side-O2	10%
Curcumin	Curcumin-Side-O5 ASP131-Side-OD1	40%
	Curcumin-Side-O3 ASP131-Side-OD2	40%
	Curcumin-Side-O3 ASP131-Side-OD1	1.20%
	VAL132-Main-N Curcumin-Side-O3	80%
Ganoderic acid	Ganoderic-Side-O7 ILE340-Main-O	10%
	Ganoderic-Side-O6 ILE340-Main-O	19%
	Ganoderic-Side-O5 PRO339-Main-O	19%
	SER341-Side-OG Ganoderic-Side-O4	19%
	SER341-Side-OG Ganoderic-Side-O5	29%

In the case of the apigenin complex, during MD, the protein-apigenin complex stability was maintained by interactions with GLY 81, LYS 105, and ILE 147 residues. It has hydrogen bonds with the highest occupancy of 11.28%, where apigenin forms the hydrogen bond with LYS 105. Then 0.90% occupancy, where apigenin plays a role as an acceptor for GLY 81, and 0.10% with ILE 147. Although the Apigenin complex has 50 hydrogen bonds more than the other complexes, the compound (Apigenin) formed only two hydrogen bonds, which were found in docked simulation. Curcumin has 28 total hydrogen bonds, with the highest occupancy of 80%, where curcumin and VAL 132 form hydrogen bonds. Then 40% occupancy, where curcumin plays a role as an acceptor of ASP 131, and 1.20% with ASP 131. Only three of the 28 hydrogen bonds were formed in the docked simulation. We conclude that, during MD, the curcumin complex has more stability than the apigenin complex. Ganoderic acid has 32 total hydrogen bonds with the highest occupancy of 29%, where Ganoderic acid has the role of a donor and SER 341 has the role of an acceptor of hydrogen bond formation. Then 19% occupancy where Fisetin plays a role as an acceptor to ILE 340 and 19% with PRO 339. Only two of the 32 hydrogen bonds were formed in the docked simulation. In the system, hydrogen bonds were stable, and most of them appeared between 0 and 15 ns and 40 and 50 ns. Hydrogen bond occupancy with a score of more than 100% indicates that more than one atom pair interacts to form hydrogen bonds. Meanwhile, curcumin has a lower total hydrogen bond but a higher hydrogen bond occupancy than Ganoderic acid. This total hydrogen bond and hydrogen bond occupancy determine the stability of each system. The number and occupancy of hydrogen bonds are the keys to the interaction stabilization of the protein-ligand complex.

#### 4.4.5. Principal component analysis (PCA) and dynamics cross-correlation matrices (DCCM) analysis

**Figure 11:**
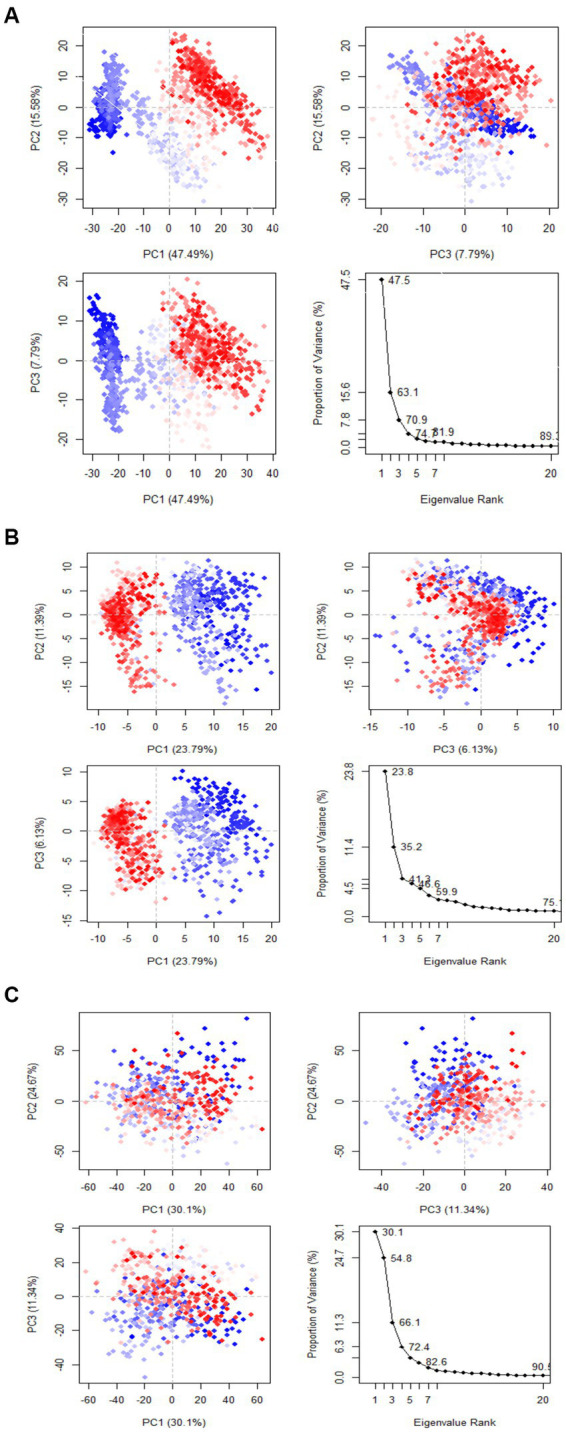
Principal component analysis of **(A)** Apigenin, **(B)** Curcumin, and **(C)** Ganoderic acid.

Principal component analysis was used to identify and understand significant concerted motions in different regions of the protein. PCA extracts the most variable dynamic motions in simulations, which are required for biological function. In addition, it may be used to examine the effect of various parameters on the collective motion and to reduce the motion’s complexity, which is related to the system’s stability and protein functions. Also, it can be utilized to characterize the many conformational variations that are associated with the process of protein folding as well as the open-close mechanism of ion channels. The conformational alterations of West Nile virus methyltransferase and West Nile virus envelope glycoprotein were further analyzed using dynamic cross correlation matrix (DCCM) analysis. Principal component analysis (PCA) and a DCCM were used to identify protein backbone conformation shifts. PCA and DCCM were performed using RStudio and Bio3d; axes, each dot represents a different protein configuration. The distribution of blue and red dots represented the extent of conformational changes in the simulation, where the color ranges from blue to white to red corresponds to simulation duration. The color blue represents the initial timestep, the color white represents the intermediate, and the color red represents the final timestep (illustrated in Figure 11).

**Figure 12:**
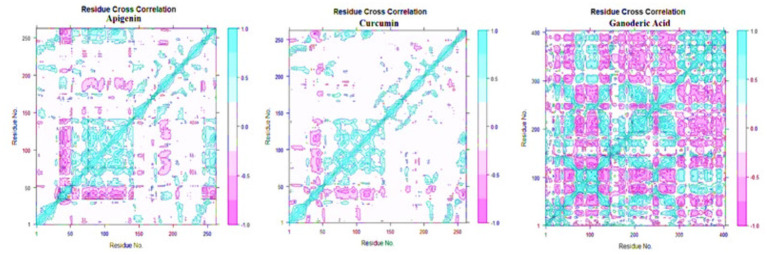
Dynamic cross correlation matrix (DCCM) plots for (Apigenin, Curcumin, and Ganoderic Acid).

The first three PCs predicted the majority of the motion of the protein backbone from the MD trajectories in Figure y. In the apigenin protein, PCA analysis shows that the first three eigenvectors account for 47.49, 47.49, and 7.79% (Figure 12A). In the case of curcumin protein, PCA analysis display that the first three eigenvectors account for 23.79, 23.79, and 6.13% (Figure 12B). For Ganoderic Acid Protein, PCA analysis display that the first three eigenvectors account for 30.1, 30.1, and 11.34% (Figure 12C). The highest PC1 (47.49%) was noticed for the apigenin protein, which indicates that the protein had undergone higher conformational changes. The lowest PC1 (6.13%) was observed for the curcumin protein, indicating that the protein had undergone very few conformational changes compared to apigenin. Comparatively, the Ganoderic acid compound exhibited less conformational change than the apigenin complex but more than the curcumin complex.

Additionally, the resulting dynamical cross-correlation graphs exhibit both positive and negative amino acid correlation effects. Overall correlation was displayed by DCCM, and it was in the range of −1.0 to 1.0. (From dark purple to dark blue). Various colors were used to denote varying degrees of association between residues, with darker colors indicating stronger correlations. Correlations closer to 1 indicated that the residues were moving in the same direction, while correlations closer to −1 indicated that the residues were moving in the opposite direction. In order to see the relationship between the I and J residue indices, we constructed pairwise correlated graphs. Colors such as dark cyan, white, and pink were used to analyze the predicted map results. Fully correlated pairs are denoted by the color cyan, while anti-correlated pairs are denoted by the color pink. Comparative results reveal that the atomic motions in the Apigenin complex resemble more closely those of the Curcumin structure. But, Comparing the DCCM diagrams of the three systems, it could be found that the correlated motions of the apigenin and curcumin systems were noticeably distinct from the Ganoderic complex. We postulate that the simultaneous appearance of positively and negatively correlated movements destabilizes the domain. For this reason, we observed high anti-correlation in the apigenin complex compared to the curcumin complex, indicating a more compact structure of the apigenin. For the Ganoderic acid complex, which showed less anticorrelated and correlated motions and more noncorrelated motions than the Apigenin and Curcumin complexes in all regions of the protein (illustrated in Figure 12).

#### 4.4.6. Binding free energy analysis

**Figure 13:**
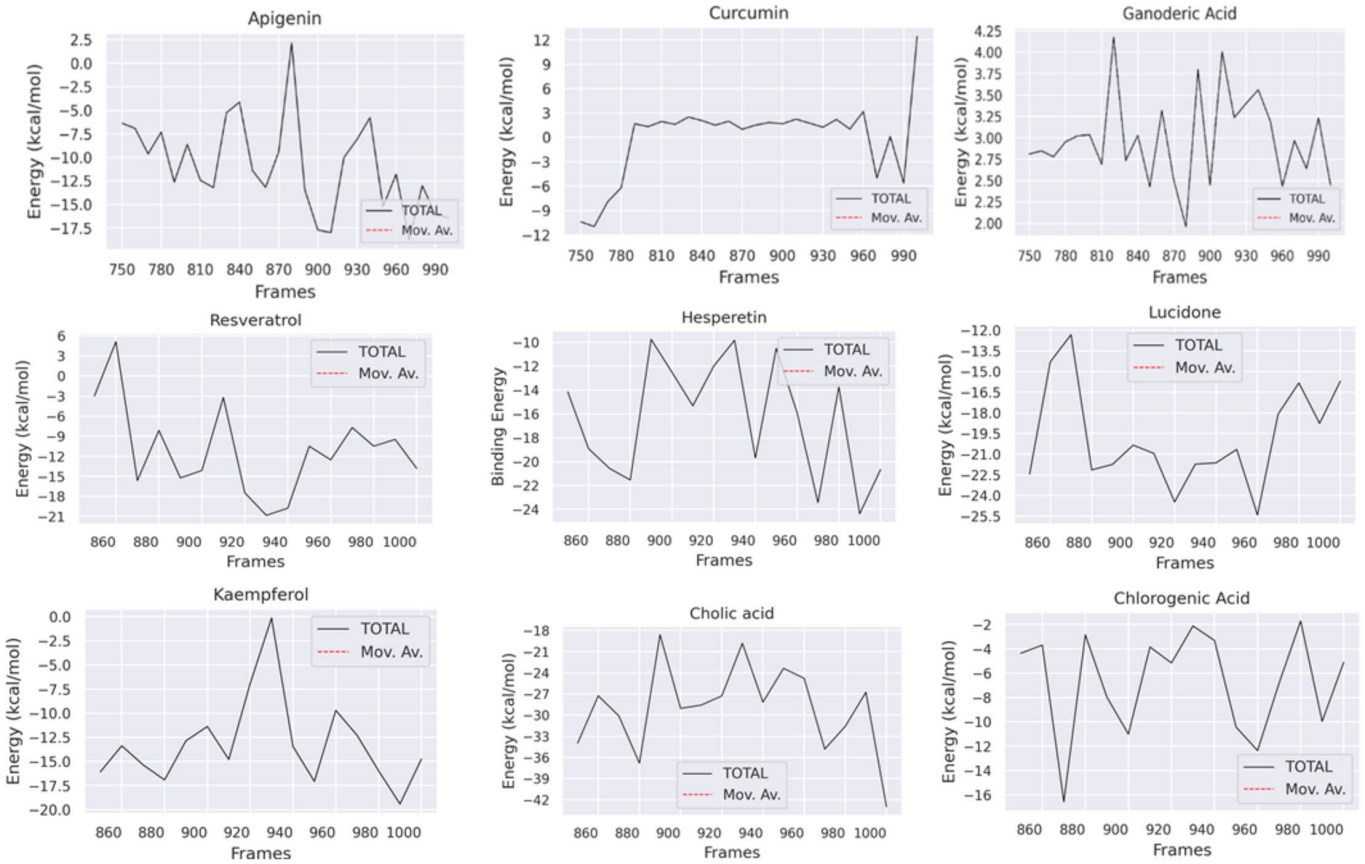
Binding free energy plot.

To analyze the molecular binding interaction of protein-ligand complexes, the binding free energy (G) was calculated using MM-PBSA, which considers both bonded and non-bonded (van der Waals and electrostatic) interactions. Using MMPBSA and the final 20 ns of the trajectory, the binding free energies of the protein-ligand complexes were calculated. Using the MMGBSA approach, the binding free energy (∆ Gbind) of 12 natural compounds, of which top nine were chosen based on binding affinity score, was determined. The greater the negative values, the more favorable the binding free energy between proteins and ligands. As shown in Table 4 and Figure 13, the free binding energies of the natural compounds agree with the molecular docking results. The Hesperetin, Lucidone, and Cholic acid demonstrated the greatest amount of binding energy (−16.44 kcal/mol, −19.78 kcal/mol and − 29.00) in comparison to the other natural chemicals. This energy is mostly different from the curcumin compound, and it means that the interaction in the compound Hesperetin, Lucidone, and Cholic acid complex is the most stable interaction during simulation, followed by the protein-compound complex. On the other hand, the compound Ganoderic acid complex has the most positive binding energy, indicating that the interaction between compound Ganoderic acid and the target protein active site is the weakest.

Table 5 and Figure 13 highlights the primary components of binding free energy for protein-ligand complexes, including van der Waals (ΔEVDW), electrostatics (ΔEEEL), a polar portion of solvation (ΔGPB), non-polar part of solvation (ΔGNP), dispersion (ΔGDISP), and binding energy (ΔG).

**Table 5:** Binding energy results.

		ΔEVDW (kJ/mol)	ΔEEEL (kJ/mol)	ΔGPB (kJ/mol)	ΔGNP (kJ/mol)	ΔGDISP (kJ/mol)	ΔG Binding (kJ/mol)
01	Apigenin	−21.76	−8.06	21.93	−2.98	0.0	−10.88
02	Resveratrol	−21.27	−19.65	32.80	−2.93	0.0	−11.05
03	Hesperetin	−25.87	−8.51	21.13	−3.19	0.0	−16.44
05	Lucidone	−25.46	−16.61	25.38	−3.09	0.0	−19.78
06	Ganoderic acid	−0.00	0.00	3.05	−0.06	0.0	2.99
07	Curcumin	−0.00	−1.43	6.26	−0.80	0.0	−0.05
08	Kaempferol	−22.78	−16.56	29.34	−3.14	0.0	−13.15
09	Cholic acid	−15.82	−277.93	267.47	−2.72	0.0	−29.00
10	Chlorogenic acid	−33.86	−38.33	69.52	−4.03	0.0	−6.71

### 4.5. ADME, aquatic and non-aquatic toxicity

By utilizing the pkCSM web server, ADMET (Absorption, Distribution, Metabolism, Excretion, and Toxicity) properties were performed. The Table 6 displays the reported compound’s ADMET values. Drug development fails in between 40 and 60% of cases as a result of inadequate ADMET characteristics. The virtual screening process should take this pharmacokinetics characteristic into consideration as a major factor. The range of the water solubility log S has been reported −2.449 to −6.618. All compounds had significant levels of intestinal absorption in humans excluding 10. The VDss level varies from −2.172 to 0.822, with a maximum Total Clearance rate of 0.653 mL/min/kg. 4,5,6,11 and 12 number compounds have the permeability to the Blood–brain barrier 1,2,3,7,8,9and 10 compounds cannot pass the Blood–brain barrier. The CYP450 A2 substrate can be inhibited by most of the compounds. The CYP450 2C9 substrate can only be inhibited by 12 specific drugs; other compounds are inactive. Both of these enzymes are crucial for the metabolism of drugs and are frequently present in the human liver. In the disposition and renal clearance of mostly cationic drug molecules, renal OCT2 substrate in the kidney plays a very significant function. Finally, they all are free from AMES toxicity, Skin Sensitization and Hepatotoxicity (Table 6).

**Table 6:** Summary of *in silico* ADMET prediction.

S/N	Absorption			Distribution		Metabolism		Excretion		Toxicity		
	Water solubility Log S	Caco-2 Permeability × 10^−6^	Human Intestinal Absorption (%)	VDss (human)	BBB Permeability	CYP450 1A2 Inhibitor	CYP450 2C9 Substrate	Total Clearance (ml/min/kg)	Renal OCT2 substrate	AMES toxicity	Skin Sensitization	Hepatotoxicity
01	−3.329	1.007	93.25	0.822	No	Yes	No	0.566	No	No	No	No
02	−3.178	1.17	90.935	0.296	No	Yes	No	0.076	No	Yes	No	No
03	−3.407	0.294	70.277	0.746	No	No	No	0.444	No	No	No	No
04	−6.818	1.205	94.757	0.29	Yes	No	No	0.565	No	No	No	No
05	−2.701	1.144	95.474	−0.125	Yes	Yes	No	0.11	No	Yes	No	No
06	−3.058	2.625	66.348	−2.172	Yes	Yes	No	−0.369	No	Yes	No	No
07	−2.892	1.643	78.45	0.011	No	Yes	No	−57.293	No	Yes	No	No
08	−3.04	0.032	74.29	1.274	No	Yes	No	0.477	No	No	No	No
09	−3.763	0.597	61.546	−0.804	No	No	No	0.653	No	No	No	No
10	−2.449	−0.84	36.377	0.581	No	No	No	0.307	No	No	No	No
11	−3.538	1.152	92.417	−0.386	Yes	Yes	Yes	0.122	No	No	No	No
12	−2.892	0.239	82.294	0.011	Yes	Yes	No	−0.416	No	Yes	No	No

## 5. Conclusion

Our *in-silico* research has been reported that all the natural molecules have better binding affinity, high solubility in aqueous system, free from hepatic toxicity, and skin sensitization, most of them are accepted by lipinski rule. Besides, different types of active amino acid are seen during the formation of drug protein complexes such as VAL-A:132, ASP-A:131, PHE-A:133, LYS-A:105, ASP-A:146, TRP-A:87, GLY-A:58, ASP. The most active compounds were reported Apigenin against *West Nile virus methyltransferase (PDB ID 2OY0), and* Fungisterol, & Sanguinarine against *West Nile virus envelope glycoprotein (PDB ID 2I69) with maximum binding affinity − 8.1.3 kcal/mol, and − 8.1 kcal/mol*. The drug-likeness properties, and the theoretical ADMET data is accepted by our reported phytocompounds. After that, their stability is confirmed by molecular dynamic simulation at 100 ns which is also confirmed that the molecules are highly stable when form protein ligands complex*. As, t*here is currently neither an antiviral medicine nor a vaccination that can treat WNV infection in people. In the course of drug discovery and repurposed research, various potential natural molecules have been studied in this investigation that capable to inhibit WNV *in silico* model; however, none of these candidates have performed it to the stage of clinical assessment. Now, further experimental studies should be conducted and determine their practical value to establish them as potential drug candidate for further use.

## Data availability statement

The original contributions presented in the study are included in the article/supplementary material, further inquiries can be directed to the corresponding author/s.

## Author contributions

SA, IB, MR, NM, SM, MI, SR, AG, SA-H, MZ, VJ, SS, JB, and RS: data collection, conceptualization, methodology, software, validation, formal analysis, investigation, resources, data curation, writing—original draft preparation, writing—review and editing, visualization, supervision, project administration, and funding acquisition. All authors contributed to the article and approved the submitted version.

## Funding

This research was supported by the Deanship of Scientific Research, Imam Mohammad Ibn Saud Islamic University (IMSIU), Saudi Arabia.

## Conflict of interest

The authors declare that the research was conducted in the absence of any commercial or financial relationships that could be construed as a potential conflict of interest.

## Publisher’s note

All claims expressed in this article are solely those of the authors and do not necessarily represent those of their affiliated organizations, or those of the publisher, the editors and the reviewers. Any product that may be evaluated in this article, or claim that may be made by its manufacturer, is not guaranteed or endorsed by the publisher.

## Footnotes

^1^ http://www.swissadme.ch/index.php

^2^ https://biosig.lab.uq.edu.au/pkcsm/prediction

^3^ https://pymol.org/2/
